# Supplementary material for: The structure-selective endonucleases GEN1 and MUS81 mediate complementary functions in safeguarding the genome of proliferating B lymphocytes
Source: eLife. 2022 Oct 3;11:e77073. doi: 10.7554/eLife.77073 (PMC9581529; doi:10.7554/eLife.77073)
Supplement: Transparent reporting form [file elife-77073-transrepform1.docx]

***eLife’s* transparent reporting form**

We encourage authors to provide detailed information *within their submission* to facilitate the interpretation and replication of experiments. Authors can upload supporting documentation to indicate the use of appropriate reporting guidelines for health-related research (see [EQUATOR Network](http://www.equator-network.org/%20)), life science research (see the [BioSharing Information Resource](https://biosharing.org/)), or the [ARRIVE guidelines](http://www.plosbiology.org/article/info:doi/10.1371/journal.pbio.1000412) for reporting work involving animal research. Where applicable, authors should refer to any relevant reporting standards documents in this form.

If you have any questions, please consult our Journal Policies and/or contact us: [editorial@elifesciences.org](mailto:editorial@elifesciences.org).

**Sample-size estimation**

- You should state whether an appropriate sample size was computed when the study was being designed
- You should state the statistical method of sample size computation and any required assumptions
- If no explicit power analysis was used, you should describe how you decided what sample (replicate) size (number) to use

Please outline where this information can be found within the submission (e.g., sections or figure legends), or explain why this information doesn’t apply to your submission:

We did not determine a suitable sample size prior to conducting the experiments as we did not know *a priori* what the effect size of the phenotype upon conditional deletion of our genes of interest would be. We sought to conduct a minimum of three independent experiments, with two mice per genotype in each experiment, totaling at least 6 animals per genotype group for our studies (barring any experimental mishap).

**Replicates**

- You should report how often each experiment was performed
- You should include a definition of biological versus technical replication
- The data obtained should be provided and sufficient information should be provided to indicate the number of independent biological and/or technical replicates
- If you encountered any outliers, you should describe how these were handled
- Criteria for exclusion/inclusion of data should be clearly stated
- High-throughput sequence data should be uploaded before submission, with a private link for reviewers provided (these are available from both GEO and ArrayExpress)

Please outline where this information can be found within the submission (e.g., sections or figure legends), or explain why this information doesn’t apply to your submission:

We state the number of experiments performed in the legend of each figure. We also detail the total number of animals (biological replicates) that were analyzed for each genotype in our experiments. For the preparation of the RNAseq libraries, we report the number and sex of the animals used in the legend of the corresponding figure. The RNAseq datasets have been deposited in GEO under the accession code GSE195734.

**Statistical reporting**

- Statistical analysis methods should be described and justified
- Raw data should be presented in figures whenever informative to do so (typically when N per group is less than 10)
- For each experiment, you should identify the statistical tests used, exact values of N, definitions of center, methods of multiple test correction, and dispersion and precision measures (e.g., mean, median, SD, SEM, confidence intervals; and, for the major substantive results, a measure of effect size (e.g., Pearson's r, Cohen's d)
- Report exact p-values wherever possible alongside the summary statistics and 95% confidence intervals. These should be reported for all key questions and not only when the p-value is less than 0.05.

Please outline where this information can be found within the submission (e.g., sections or figure legends), or explain why this information doesn’t apply to your submission:

Statistical analyses employed in each experiment are detailed in the figure legends. We reported the exact p-values in each figure whenever possible and plotted the 95% confidence intervals for all relevant graphs. For the analysis of the mRNA-Seq dataset, the pipeline is described in the ‘Materials and Methods’ section.

(For large datasets, or papers with a very large number of statistical tests, you may upload a single table file with tests, Ns, etc., with reference to sections in the manuscript.)

**Group allocation**

- Indicate how samples were allocated into experimental groups (in the case of clinical studies, please specify allocation to treatment method); if randomization was used, please also state if restricted randomization was applied
- Indicate if masking was used during group allocation, data collection and/or data analysis

Please outline where this information can be found within the submission (e.g., sections or figure legends), or explain why this information doesn’t apply to your submission:

For *in vivo* analysis, the mice were analyzed randomly without sorting by genotype. For *ex vivo* studies excluding the preparation of metaphase spreads, primary cell cultures were prepared and analyzed by genotype groups. Samples for the metaphase spreads and telomere FISH studies were prepared in a single-blinded and randomized manner.

**Additional data files (“source data”)**

- We encourage you to upload relevant additional data files, such as numerical data that are represented as a graph in a figure, or as a summary table
- Where provided, these should be in the most useful format, and they can be uploaded as “Source data” files linked to a main figure or table
- Include model definition files including the full list of parameters used
- Include code used for data analysis (e.g., R, MatLab)
- Avoid stating that data files are “available upon request”

Please indicate the figures or tables for which source data files have been provided:

Figure 4

Figure 4–figure supplement 1A

Figure 4–figure supplement 1B

Figure 4–figure supplement 1C
